# Beneficial effects of 20(S)-protopanaxadiol on antitumor activity and toxicity of cyclophosphamide in tumor-bearing mice

**DOI:** 10.3892/etm.2012.820

**Published:** 2012-11-20

**Authors:** GUANGZHU LIN, XIAOFENG YU, JING WANG, SHAOCHUN QU, DAYUAN SUI

**Affiliations:** 1 Department of Pharmacology, School of Pharmacy; China-Japan Union Hospital, Jilin University, Changchun 130021, P.R. China; 2 Department of Cardiovascular Medicine, First Hospital; China-Japan Union Hospital, Jilin University, Changchun 130021, P.R. China; 3 Department of Respiratory Medicine, China-Japan Union Hospital, Jilin University, Changchun 130021, P.R. China

**Keywords:** 20(S)-protopanaxadiol, cyclophosphamide, white blood cell count, spleen index

## Abstract

20(S)-protopanaxadiol (PPD) is an extract of *Panax quinquefolius* L. The aim of this study was to investigate the effect of PPD on the antitumor activity and toxicity of cyclophosphamide (CTX) in tumor-bearing mice. C57BL/6 mice bearing Lewis lung carcinoma cells were treated with PPD (50 mg/kg) alone, CTX (20 mg/kg) alone or PPD (50 mg/kg) in combination with CTX (20 mg/kg), respectively. The results showed that PPD alone has no significant antitumor activity but synergistically enhanced the antitumor activity of CTX. PPD significantly increased the peripheral white blood cell count, bone marrow cell count, interleukin-2 and interferon-γ in CTX-treated tumor-bearing mice. The lowered levels of spleen index, splenocyte proliferation and natural killer cell activity in tumor-bearing mice following CTX treatment were also increased by PPD administration. PPD may be a beneficial supplement during CTX chemotherapy for enhancing the antitumor efficacy and reducing the toxicity of CTX.

## Introduction

Cancer is one of the leading causes of mortality worldwide. Chemotherapy is one of the major therapeutic modalities commonly used for the treatment of a variety of cancer types. However, in numerous cases, chemotherapy cannot achieve a satisfactory therapeutic outcome, namely the complete remission of tumors, and induces severe side-effects at therapeutically effective doses. Cyclophosphamide (CTX) has been used widely in chemotherapy since the late 1950s and has been shown to have a high therapeutic index and broad spectrum of activity against a variety of types of cancer (1). However, the use of CTX as an effective chemotherapeutic agent is often restricted due to its wide toxicity and adverse side-effects, which include leukopenia, myelosuppression and immunosuppression (2,3). CTX is presently being used in combination with various detoxifying and protective agents with the purpose of reducing or eliminating its adverse toxic effects.

*Panax quinquefolius* L. has been used worldwide for thousands of years in traditional herbal medicine (4). Ginsenosides are considered to be one of main bioactive constituents of *Panax quinquefolius* L. and have been used clinically for the treatment of cardiovascular diseases and stroke in China (5). The anticancer properties of *Panax quinquefolius* L. and/or ginsenosides have been well documented *in vitro* and *in vivo*(6–8). Upon oral consumption, *Panax quinquefolius* L. or ginsenosides are partly transformed into 20(S)-protopanaxadiol (PPD) through a series of deglycosylations by acid hydrolysis and intestinal bacterial actions (9). PPD-type ginsenosides are generally considered to be the most pharmacologically active components of *Panax quinquefolius* L. At present, the valuable information concerning the pharmacological and toxic effects of PPD combined with chemotherapeutic agents is scarce. The present study aimed to evaluate whether PPD is able to exert beneficial effects on antitumor activity and toxicity of CTX in tumor-bearing mice

## Materials and methods

### Preparation of PPD

**Figure 1.**
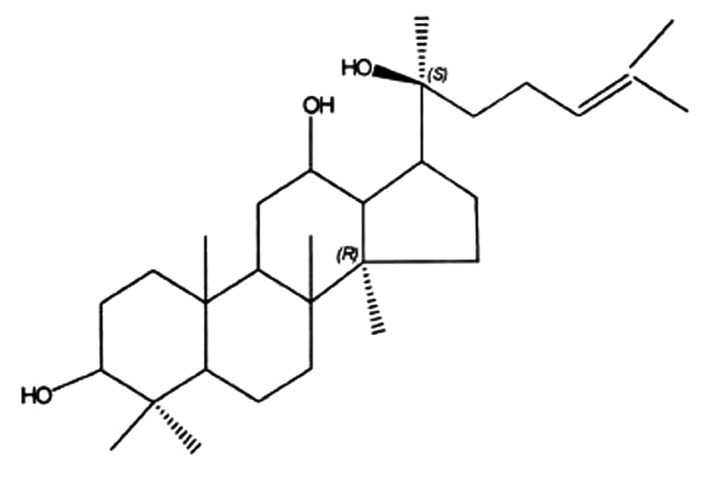
Chemical structure of 20(S)-protopanaxadiol.

PPD is extracted from protopanaxadiol Rh_2_ (Hainan Asia Pharmaceutical Co. Ltd., Haikou, China) that is prepared from the roots and leaves of *Panax quinquefolium* L. by the alkali hydrolysis of glucose at the C-3 position of the dammarane structure at 25°C. At normal pressure, the reaction medium, 4-butanediol caused the reaction temperature to increase to 180°C in alkali solution. The PPD was purified by silica gel column chromatography and acetic ether re-crystallization. The yield of the extract as a dried material was ∼1.8% by weight of the original material. Further analysis by HPLC showed that the content of PPD in the resultant extract was 98.6% (Fig. 1).

### Animals

Male C57BL/6 mice (7–8 weeks old) were purchased from the Institute of Zoology of the Chinese Academy of Sciences (Beijing, China) and the certificate number was SCXK11-00-0006. The mice were acclimated to laboratory conditions (22±2°C) and (55±5% humidity) for 7 days, with a commercial standard mouse cube diet (Experimental Animal Center of Jilin University, Changchun, China) and water *ad libitum* prior to the experiment. All animal experiments were conducted in compliance with the National Institute of Health Guidelines for the Care and Use of Laboratory Animals (publication 86-23, revised in 1986) and were approved by the local Ethics Committee.

### Materials

The culture medium RPMI-1640 and fetal calf serum were from Gibco (Grand Island, NY, USA). The concanavalin A was purchased from Sigma Chemical Co. (St. Louis, MO, USA). The penicillin and streptomycin were from Huabei Pharmaceutical Co. Ltd. (Shijiazhuang, China). Lewis lung carcinoma (LLC) cells were purchased from the China Center for Type Culture Collection (Beijing, China). The CTX was provided by the Jiangsu Hengrui Company (Jiangsu, China). The ELISA kits of interleukin-2 (IL-2) and interferon-γ (INF-γ) were purchased from Westang Biomedical Technology Company (Shanghai, China).

### Treatment and drug administration

LLC cells were cultured in RPMI-1640 complete medium with 10% heat-inactivated FBS. LLC cells (0.2 ml, 1×10^7^ cells/ml in sodium chloride) were implanted subcutaneously into the mice. After implantation for 24 h, the mice bearing LLC cells were randomly divided into four groups with 10 mice in each: the control group (Control), PPD (50 mg/kg) alone group (PPD), CTX (20 mg/kg) alone group (CTX) or PPD (50 mg/kg) in combination with CTX (20 mg/kg) group (PPD+CTX). PPD suspended in saline was orally administered once a day for 14 consecutive days. CTX dissolved in saline was intraperitoneally injected once a day on days 1, 3, 5 and 7 (total 4 injections) at a dose of 20 mg/kg body weight. The mice in the PPD+CTX group received orally administered PPD (50 mg/kg) once a day for 14 consecutive days and were intraperitoneally injected with CTX once a day on days 1, 3, 5 and 7. The mice in the control group received saline alone (20 ml/kg).

### Antitumor activity of PPD in combination with CTX

On day 15, the mice were anesthetized with sodium pentobarbital (300 mg/kg, intraperitoneally) and were sacrificed by cervical dislocation. The implanted sarcomas of each group were then separated and weighed.

### Peripheral white blood cell and bone marrow cell counts

Blood and serum samples and femur bones were obtained from the tumor-bearing mice. Blood was collected from the retroorbital venous plexus in heparinized tubes. Bone marrow was collected from the left femur bones by flushing thoroughly with Hank’s balanced solution using a 28-gauge needle in a 1-ml syringe. The cells were collected in a sterile tube and diluted to a total volume of 5 ml. Peripheral white blood cell (WBC) and bone marrow cell (BMC) counts were evaluated microscopically using a hematocytometer (ABX micros 60; Horiba, Montpellier, France). The serum IL-2 and INF-γ levels were assayed using commercial reagent kits.

### Spleen index and splenocyte proliferation assay

After the last drug administration (24 h), the mice were weighed and sacrificed by cervical dislocation. The spleens of the mice were removed and weighed under sterile conditions. The spleen index was calculated as spleen weight (mg)/body weight (g). The fresh spleen sample was used to evaluate the splenocyte proliferation. Splenocytes from the tumor-bearing mice were prepared as previously described (10) and seeded into 96-well flat-bottom microplates at 1×10^6^ cell/ml in 100 μl complete medium. Subsequently, concanavalin A (final concentration 5 μg/ml) or RPMI-1640 medium were added, giving a final volume of 200 μl. The plates were incubated at 37°C in a humid atmosphere with 5% CO_2_. After 44 h, 50 μl of MTT solution (2 mg/ml) were added to each well and incubated for further 4 h. The plates were centrifuged (1400 x g, 5 min) and the untransformed MTT was removed carefully by pipetting. To each well, 150 μl of a DMSO working solution (180 μl DMSO with 20 μl 1 M HCl) was added and the absorbance was evaluated using an ELISA reader (Synergy HT; BioTek, Winooski, VT, USA) at 570 nm after 15 min. The splenocyte proliferation was calculated based on the following formula: splenocyte proliferation = absorbance value of concanavalin A-stimulated cultures / absorbance value of non-stimulated cultures.

### Natural killer (NK) cell activity

Splenocytes were prepared as the effector cells for the splenic NK cell activity assay as described previously (11). YAC-1 cells were used as the target cells. Briefly, effector cells (5×10^5^ cells/well) in 96-well flat-bottom microplates were co-cultured in triplicate with target cells at 37°C in a humid atmosphere of 5% CO_2_ at a ratio of effector to target cells of 50:1. Serum-free RPMI-1640 medium was used as a control. The NK cell activity of the splenocytes was measured using the MTT assay after 24 h of culture. The MTT solution (5 mg/ml) was added to each well. After 4 h of incubation, the cells were lysed and the purple formazan crystals were solubilized by DMSO for detection at 570 nm using a microplate reader. The NK activity of the effector cells was calculated using the following formula: cytotoxicity (%) = (A+B−C) / A×100, where A is the absorbance of the well of target cells, B is the absorbance of the well of effector cells and C is the absorbance of the experimental well.

### Statistical analysis

The data were presented as mean ± standard deviation (SD). Data were analyzed by one-way analysis of variance (ANOVA), followed by Student-Newman-Keuls tests. P<0.05 was considered to indicate statistically significant differences.

## Results

### Effects of PPD on antitumor activity in CTX-treated tumor-bearing mice

**Figure 2.**
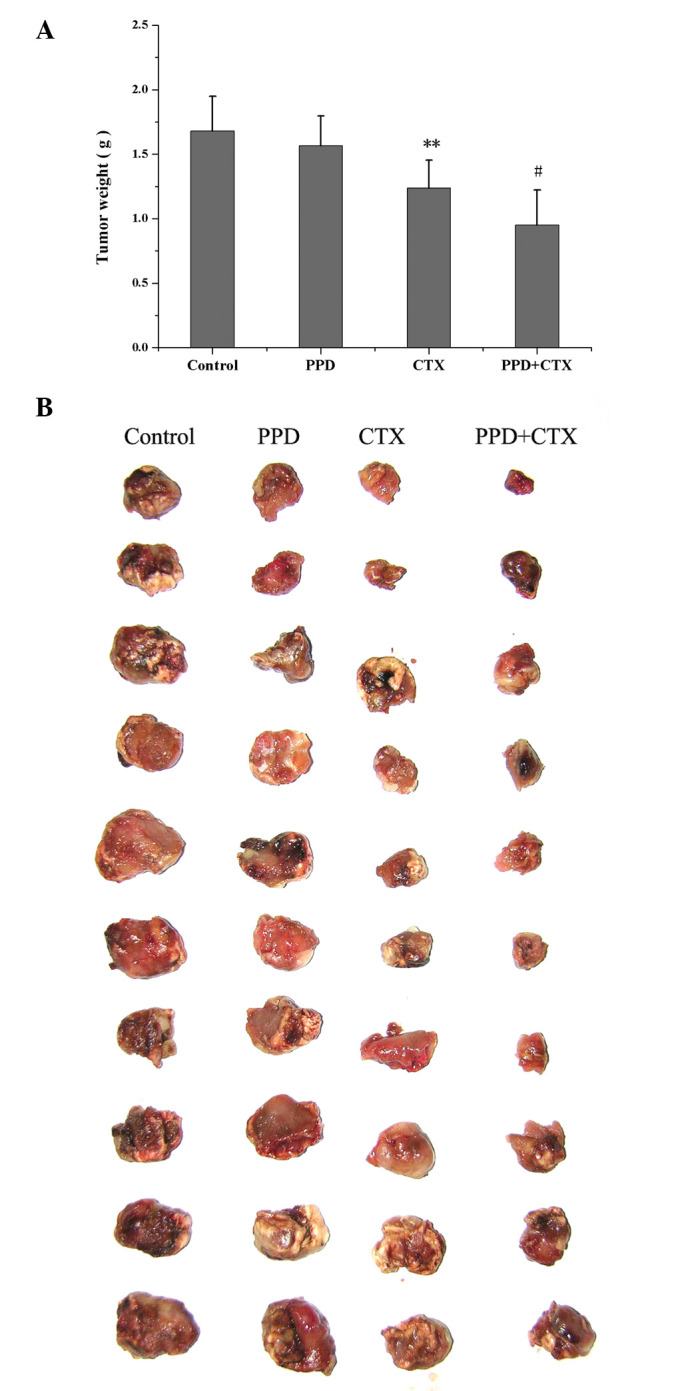
(A) Effects of PPD on antitumor activity in CTX-treated tumor-bearing mice. Data are expressed as the mean ± SD (n=10). Statistical significance was determined using ANOVA, followed by Student-Newman-Keuls tests. ^**^P<0.01 compared with the control group; ^#^P<0.05 compared with the CTX group. (B) Images of excised tumors at the time of sacrifice from the subcutaneous tumor-bearing mice after PPD, CTX or PPD in combination with CTX treatment. PPD, 20(S)-protopanaxadiol; CTX, cyclophosphamide; ANOVA, analysis of variance.

The effects of PPD on CTX-induced changes of tumor weight in tumor-bearing mice are shown in Fig. 2. Compared with the control group, PPD alone had no effects on tumor weight, while CTX alone significantly reduced tumor weight (P<0.01). PPD in combination with CTX significantly decreased tumor weight (P<0.05).

### Effects of PPD on WBC and BMC counts in CTX-treated tumor-bearing mice

**Figure 3.**
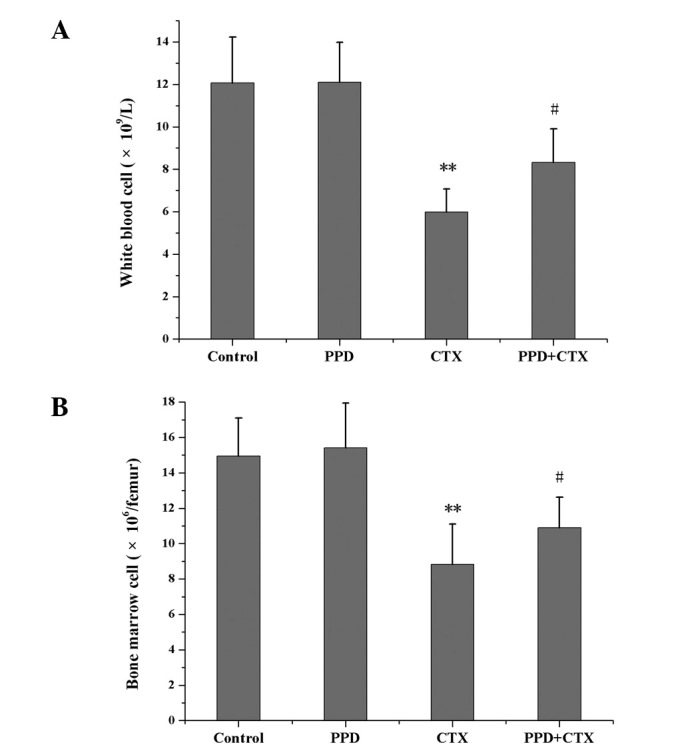
Effects of PPD on (A) peripheral white blood cell and (B) bone marrow cell counts in CTX-treated tumor-bearing mice. Data are expressed as the mean ± SD (n=10). Statistical significances were determined using ANOVA, followed by Student-Newman-Keuls tests. ^**^P<0.01 compared with the control group; ^#^P<0.05 compared with the CTX group. PPD, 20(S)-protopanaxadiol; CTX, cyclophosphamide; ANOVA, analysis of variance.

Compared with the control group, PPD alone had no effects on WBC and BMC counts, while CTX alone reduced WBC and BMC (P<0.01) counts. However, compared with the CTX group, PPD in combination with CTX significantly increased WBC and BMC counts (P<0.05; Fig. 3).

### Effects of PPD on spleen index and splenocyte proliferation in CTX-treated tumor-bearing mice

**Figure 4.**
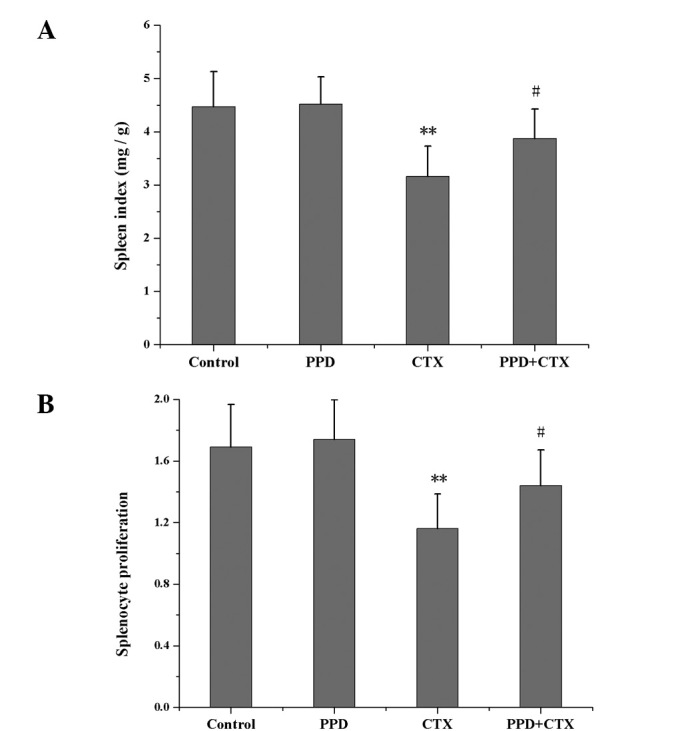
Effects of PPD on (A) spleen index and (B) splenocyte proliferation in CTX-treated tumor-bearing mice. Data are expressed as the mean ± SD (n=10). Statistical significances were determined using ANOVA, followed by Student-Newman-Keuls tests. ^**^P<0.01 compared with the control group; ^#^P<0.05 compared with the CTX group. PPD, 20(S)-protopanaxadiol; CTX, cyclophosphamide; ANOVA, analysis of variance.

As shown in Fig. 4, the spleen index and splenocyte proliferation values of the CTX-treated group were much lower than those of the control group (P<0.01). Treatment with PPD in combination with CTX increased the spleen index and splenocyte proliferation in tumor-bearing mice (P<0.05).

### Effects of PPD on NK cell activity in CTX-treated tumor-bearing mice

**Figure 5.**
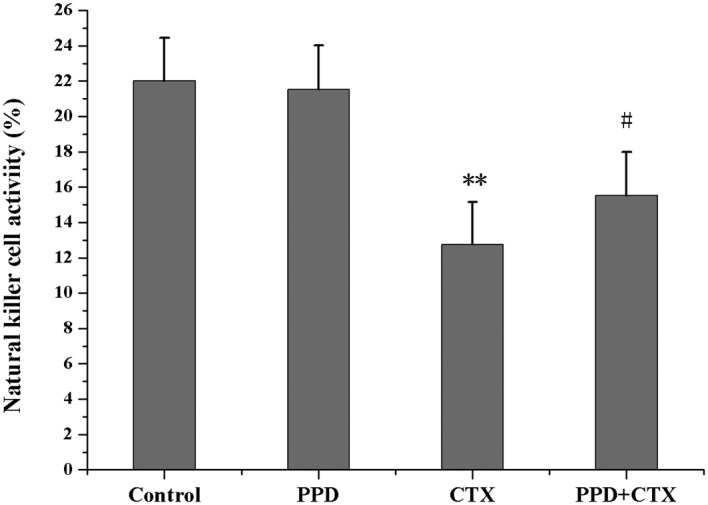
Effects of PPD on NK cell activity in CTX-treated tumor-bearing mice. Data were expressed as the mean ± SD (n=10). Statistical significances were determined using ANOVA, followed by Student-Newman-Keuls tests. ^**^P<0.01 compared with the control group; ^#^P<0.05 compared with the CTX group. PPD, 20(S)-protopanaxadiol; CTX, cyclophosphamide; ANOVA, analysis of variance.

As shown in Fig. 5, NK cell activity in CTX-treated tumor-bearing mice was markedly decreased when compared with the control group (P<0.01). However, NK cell activity in the tumor-bearing mice co-treated with PPD and CTX was significantly higher than that of mice receiving CTX treatment alone (P<0.05).

### Effects of PPD on the levels of IL-2 and INF-γ in CTX-treated tumor-bearing mice

**Figure 6.**
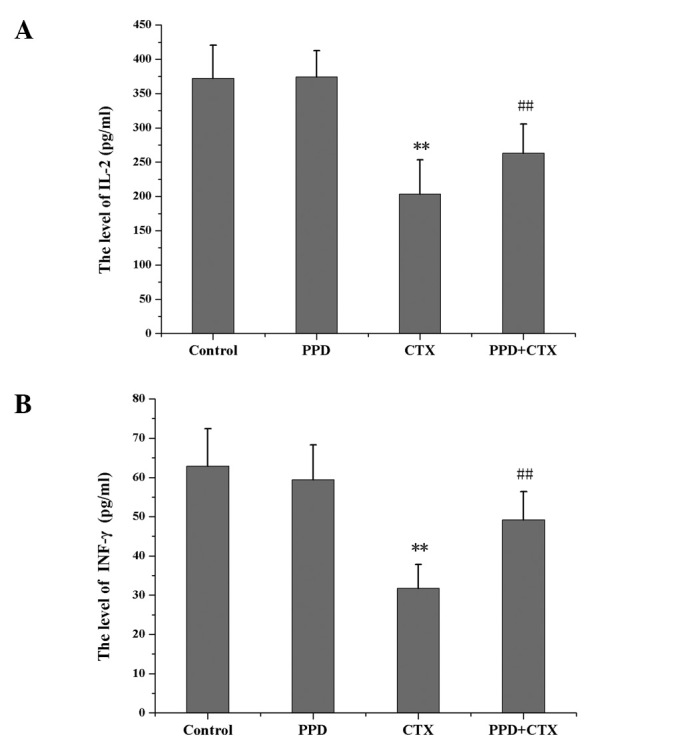
Effects of PPD on the levels of (A) IL-2 and (B) INF-γ in CTX-treated tumor-bearing mice. Data were expressed as the mean ± SD (n=10). Statistical significances were determined using ANOVA, followed by Student-Newman-Keuls tests. ^**^P<0.01 compared with the control group; ^#^P<0.05 compared with the CTX group. PPD, 20(S)-protopanaxadiol; CTX, cyclophosphamide; IL-2, interleukin-2; INF-γ; interferon-γ ANOVA, analysis of variance.

IL-2 and INF-γ levels were significantly decreased in CTX-treated tumor-bearing mice as compared with those of the control group (P<0.01). Compared with the CTX group, the IL-2 and INF-γ levels in the PPD+CTX group were significantly increased (P<0.01; Fig. 6).

## Discussion

Although CTX is a drug widely applied in the treatment of malignant and nonmalignant tumors, the clinical outcomes of treatments with these agents are severely limited, mostly due to its toxicity to normal tissues. Therefore, it is necessary to develop adjuvant therapy which may be used in combination with CTX to improve the efficacy of the treatment or reduce the associated undesirable side-effects (12). The main objective of this study was to evaluate the effect of PPD on the antitumor activity and toxicity of CTX in tumor-bearing mice.

Following an administration of PPD alone to LLC-bearing mice, the tumor weight was not reduced. By contrast, the combination of PPD with CTX significantly reduced the tumor weight when compared with that of the control and CTX alone groups. Thereby, it is possible to conclude that PPD is able to enhance antitumor activity of CTX.

The peripheral WBC and BMC counts are two frequently studied clinical parameters which accurately reflect chemotherapeutic injuries. In order to study the effect of PPD on CTX-induced leukopenia and myelosuppression, the peripheral WBC and BMC counts in CTX-treated LLC-bearing mice were measured. The results showed that PPD significantly recovered the reduced WBC and BMC counts in LLC-bearing mice treated with CTX, suggesting that PPD may provide preferential protection against leukopenia and myelosuppression induced by CTX.

The spleen is one of the immune organs that generates immune cells, such as lymphocytes and macrophages, which phagocytose and destroy bacteria and dead tissue in order to remove them from the circulating blood (13). The most sensitive indicator of immunosuppression, particularly in short-term studies, is a decrease in the relative spleen weight. The proliferation of splenocytes is known to be a response to stimulation induced by antigens or mitogens, which is a typical non-specific immune reaction with a well-understood mechanism. Moreover, this assay has been extensively used as an immune parameter to investigate lymphocyte responsiveness due to its high sensitivity. In the present experiment, CTX not only caused spleen atrophy but also decreased splenocyte proliferation. However, the results showed that treatment with PPD inhibited spleen atrophy and promoted the recovery of splenocyte proliferation in CTX-treated tumor-bearing mice.

The NK cell is an important part of the innate immune system and is key to the first-line defense against malignancies. Therefore, the use of NK cells in human cancer immunotherapy has been suggested and treatments using these cells have recently entered clinical trials (14). A number of treatment strategies have also been exploited to activate endogenous NK cells, promote NK cell proliferation or induce more potent NK cell-mediated antitumor responses (15). One major strategy is the systemic administration of cytokines involved in NK cell differentiation and activation, such as IL-2 and interferons (16). Cytokines regulate the innate immune system and increase NK cell activity. NK cells also regulate the adaptive immune system and responses to produce cytokines (17). Cytokines have been used successfully to treat several human cancers through the direct or indirect activation of NK cells (18). IL-2 is an autocrine growth factor from T lymphocytes and the transcription of IL-2 is an important step in T cell activation. IFN-γ is produced predominantly by T lymphocytes and NK cells following activation with immune and inflammatory stimuli rather than viral infection (19). The present findings showed that CTX caused significant decreases in NK cell activity and the levels of IL-2 and IFN-γ, which are consistent with previous studies (20). However, PPD significantly increased the NK cell activity and levels of IL-2 and IFN-γ, suggesting that PPD may improve cellular immune function in CTX-treated tumor-bearing mice.

In summary, the results of the present study demonstrated that PPD synergistically enhanced the antitumor activity of CTX. PPD significantly increased WBC count, BMC count and the levels of IL-2 and IFN-γ in CTX-treated tumor-bearing mice. The lowered levels of spleen index, splenocyte proliferation and NK cell activity in tumor-bearing mice following CTX treatment were also increased by PPD administration. Therefore, PPD may be a beneficial supplement during CTX chemotherapy to enhance the antitumor efficacy and reduce the toxicity of CTX.
